# Transcriptional Regulation of Flotillins by the Extracellularly Regulated Kinases and Retinoid X Receptor Complexes

**DOI:** 10.1371/journal.pone.0045514

**Published:** 2012-09-18

**Authors:** Antje Banning, Wymke Ockenga, Fabian Finger, Philipp Siebrasse, Ritva Tikkanen

**Affiliations:** Institute of Biochemistry, Medical Faculty, University of Giessen, Giessen, Germany; Hungarian Academy of Sciences, Hungary

## Abstract

Flotillin-1 and flotillin-2 are important regulators of signal transduction pathways such as growth factor signaling. Flotillin expression is increased under pathological conditions such as neurodegenerative disorders and cancer. Despite their importance for signal transduction, very little is known about the transcriptional regulation of flotillins. Here, we analyzed the expression of flotillins at transcriptional level and identified flotillins as downstream targets of the mitogen activated kinases ERK1/2. The promoter activity of flotillins was increased upon growth factor stimulation in a MAPK dependent manner. Overexpression of serum response factor or early growth response gene 1 resulted in increased flotillin mRNA and protein expression. Furthermore, both promoter activity and expression of endogenous flotillins were increased upon treatment with retinoic acid or by overexpression of the retinoid X receptor and its binding partners RARα and PPARγ. Our data indicate that the expression of flotillins, which can be detected in all cultured cells, is fine-tuned in response to various external stimuli. This regulation may be critical for the outcome of signaling cascades in which flotillins are known to be involved.

## Introduction

The family of flotillin proteins consists of two homologous members, flotillin-1 and flotillin-2, also termed “reggies”, with flotillin-1 being identical to reggie-2 and flotillin-2 to reggie-1 [1], [2]. Flotillins are highly conserved among species and show a wide expression pattern in various cell and tissue types (see overview in [3]). Flotillins are constitutively associated with certain types of membrane microdomains known as “lipid rafts” by means of acylation [4], [5] and oligomerization [6], [7], and they do not traverse the membrane but reside on the cytoplasmic side of it.

One major function of lipid rafts is to organize signaling partners into functional complexes [8]. Within rafts, flotillins have been suggested to provide platforms for the assembly of signaling molecules and thus function as regulators of several signal transduction pathways associated with membrane receptors, e.g. insulin [9], IgE receptor [10], IL-6/STAT3 signaling [11], G protein coupled receptor [12] and of the neurotrophin receptor TrkA. Furthermore, we have recently shown that flotillin-1 is crucial for receptor tyrosine kinase signaling through the epidermal growth factor (EGF) [13] and fibroblast growth factor (FGF) receptors [14] and thereby regulates the MAP kinase signaling [13]. In the case of all signal transduction pathways in which flotillins are known to be involved, the absence of flotillins leads to a severe impairment of the signaling cascade [10]–[13]. During EGF receptor (EGFR) signaling, the absence of flotillin-1 results in reduced phosphorylation of specific tyrosines in the EGFR and in inefficient activation of the downstream mitogen activated protein (MAP) kinase and Akt signaling [13]. The cellular activation ERK1/2 is preceded by the activation of receptor tyrosine kinases (e.g. EGFR) by growth factors, which is followed by the binding of the SH2 domain-containing adaptor protein Grb2 in association with the guanine exchange factor SOS, activation of Ras and subsequently of the serine-threonine kinase Raf. Active Raf kinase phosphorylates MEK1/2 which in turn phosphorylate ERK1/2 on both tyrosine and threonine residues.

Despite their almost ubiquitous expression, information on the regulation of flotillin expression is scarce. At protein level, flotillins stabilize each other, and siRNA-mediated knockdown of flotillin-2 severely reduces the expression of flotillin-1, while the mRNA level is not affected ([7] and our own unpublished data). In HeLa cells, flotillin-1 depletion results in about 50% loss of flotillin-2, again without effect on the mRNA level [13]. Heterooligomerization with flotillin-2 appears to be an important factor for stabilizing flotillin-1 and enabling the endocytosis of flotillins after growth factor stimulation [6].

Flotillin expression is altered under some pathological conditions. In 60% of human breast cancer samples, flotillin-1 was found to be overexpressed, which correlates with a poor patient survival [15], and in renal cell carcinomas [16]. Similarly, flotillin-2 is upregulated in melanoma [17], [18] and in metastatic nasopharyngeal carcinoma cells [19]. Moreover, flotillin expression is increased in neurodegenerative diseases such as Alzheimer's disease [20]–[22], and Parkinson's disease [23], as well as in severe acute respiratory syndrome (SARS) [24]. Overexpression of flotillin-2 in non-tumorigenic melanoma cells results in melanoma progression and formation of metastases [18].

Because of their ability to modulate the outcome of signaling cascades and to stimulate cell proliferation [15], [18], [25], cell spreading [26] and filopodia formation [4], [27], which are processes important during carcinogenesis and metastasis formation, the correct cellular amount of flotillins is crucial for the ability of a cell to react on certain external stimuli (e.g. growth factors) and to maintain a balance between cell proliferation and cell death. Thus, the regulation of the expression of flotillins in order to allow for the cells to adapt to environmental clues is of major importance. Nevertheless, very little information is available on factors that regulate flotillins at transcriptional level. Flotillin-2 was described to be a target gene of the p53 transcription factor family members p63 and p73, but not of p53 itself [11]. Lopez-Casas *et al*. [28] showed that flotillin-1 mRNA is upregulated upon increasing cell density in 3T3 fibroblasts. They isolated a fragment of the promoter of murine flotillin-1 and identified putative binding sites for Sp1, AmL-1a, MZF1, c-Ets1, and Lyf-1 in the genomic sequence within about 500 bp upstream of the transcription start site. Unfortunately, the functional role of none of these transcription factors in the regulation of flotillin-1 transcription was analyzed further. In microarray experiments, both flotillin-1 and flotillin-2 were found to be upregulated upon stimulation of myeloid progenitor cells with granulocyte colony-stimulating factor (G-CSF), a cytokine that leads to differentiation of progenitor cells into neutrophilic granulocytes in a STAT3-dependent process [29]. In drosophila, flotillin-2 has recently been shown to be transcriptionally regulated upon embryonic wound response [30].

**Table 1:** Oligonucleotides and plasmids.

Oligonucleotide or plasmid	Sequence (5′→3′)^a^ or description (Restriction sites are underlined)	Accession no. or reference
**Oligos**		
Flot1-2660 fwd	GAGGTACCCTGTGGCTAAAGTGAGCCCCTC (KpnI)	
Flot1-1330 fwd	ACCTGAAGCTTCGGCAAAGTAGGAG (HindIII)	
Flot1-630 fwd	CCAGGTACCCCGGCTGGGCCCGAGAGCCAG (KpnI)	
Flot1-375 fwd	CCGAGAAGCTTGCCGGGCTGCCGCC (HindIII)	
Flot1 rev	AAAAGCTTGTTCAGGCTGGAGCTGGAGGAG (HindIII)	
Flot2-2130 fwd	CAGAGGTACCTGGTGTGCTTGAGAAAGGCT (KpnI)	
Flot2-1385 fwd	TAGCTAAGCTTGTAAATACACACCA (HindIII)	
Flot2-930 fwd	TCTAAGAAGCTTCACCGAATTCAGG (HindIII)	
Flot2-560 fwd	AGAGGTACCGAGTTTGCAATGTGATGCACT (KpnI)	
Flot2 rev	TTAAGCTTGGCGCCGGCGGCACGGAGGGCC (HindIII)	
Flot2 ChIP fwd	GAGCTAAGGGACTCGCCTAG	
Flot2 ChIP rev	CTCGGGACCGCACAGACCCG	
Flot1 mouse fwd	GCTTCTCAGGGGCAGCTGCA	
Flot1 mouse rev	CACAAGTGAAAAGCTTGGTTGAAGC	
Flot2 mouse fwd	GCATTGAGCCCCTCAGCGGGA	
Flot2 mouse rev	TGGCAGTTAAGCTTGGCGCCGTTGGCA	
mFlot2-dEgr1	CTATA AAGCTT GCCCCGCCGGAAGTGTAGCG	
RXR1-mut-fwd	GCTAGAGATGACCAGGCTGCTCAA**TAATA**ATGGAAAGCTACCAAGAAGTTGG	
RXR1-mut-rev	CCAACTTCTTGGTAGCTTTCCAT**TATTA**TTGAGCAGCCTGGTCATCTCTAGC	
RXR2-mut-fwd	GAACCTACACCCTAACTTTCC**TTTAA**CCCGGGATAGAATGC	
RXR2-mut-rev	GCATTCTATCCCGGG**TTAA**AGGAAAGTTAGGGTGTAGGTTC	
RXR3-mut-fwd	GGGTTGCCACATTTACCTCGG **TTAA** TCTTGTCATGTCCTGAACAGCCC	
RXR3-mut-rev	GGGCTGTTCAGGACATGACAAGA**TTAA**CCGAGGTAAATGTGGCAACCC	
qPCR-Rpl13a fwd	CCTGGAGGAGAAGAGGAAAGAGA	NM_012423.2
qPCR-Rpl13a rev	TTGAGGACCTCTGTGTATTTGTCAA	NM_012423.2
GAPDH fwd	CATCTTCCAGGAGCGAGATCCC	NM_002046.3
GAPDH rev	CCAGCCTTCTCCATGGTGGT	NM_002046.3
qPCR-Flot1 fwd	TATGCAGGCGGAGGCAGAAG	NM_005803.2
qPCR-Flot1 rev	CAGTGTGATCTTATTGGCTGAA	NM_005803.2
qPCR-Flot2 fwd	GAGATTGAGATTGAGGTTGTG	NM_004475.2
qPCR-Flot2 rev	ATCCCCGTATTTCTGGTAGG	NM_004475.2
**Plasmids**		
pGL3-basic	Firefly luciferase reporter gene plasmid without any promoter	Qiagen
pRL-TK	Renilla luciferase reporter gene plasmid for normalization	Promega
pCIG-VP16 RARα	RARα expression plasmid	Addgene 16287, [81]
pSV Sport RXRα	RXRα expression plasmid	Addgene 8882, [82]
pcDNA3-Egr1	Egr-1 expression plasmid	Addgene 11729, [36]
pCGN-SRF	SRF expression plasmid	Addgene 11977, [83]
pSV Sport PPARγ	PPARγ expression plasmid	Addgene 8886, [82]
pSV Sport	Control expression plasmid	Invitrogen
WT-ERK2	Wild-type rat ERK2	[32], N. Ahn
CA-ERK2	Constitutively active ERK2 (Leu72Pro+Ser151Asp)	[32], N. Ahn
DN-ERK2	Dominant negative ERK2 (Lys52Arg)	[32], N. Ahn

In order to characterize the signals that modulate the expression level of flotillins, we have here analyzed the proximal promoter regions of human and murine flotillins. We here show that the transcription factors Egr1, SRF, RXRα, RARα, and PPARγ are positive regulators of flotillin-1 and flotillin-2 expression and that several stimuli that activate the MAP kinase pathway result in increased expression of flotillins.

**Figure 1:**
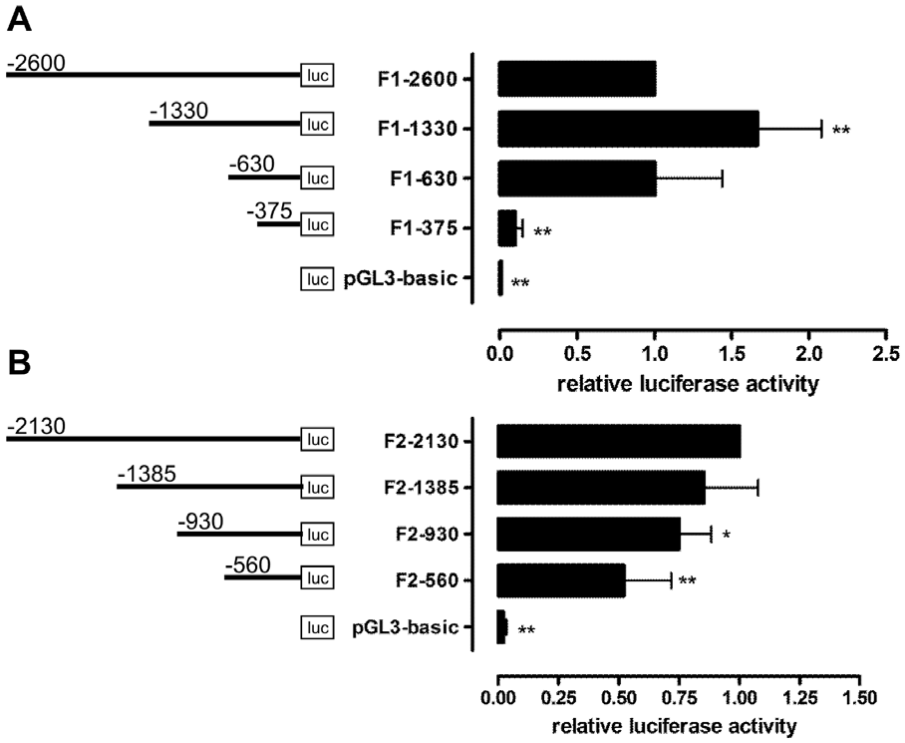
Basal activity of flotillin-1 (A) and flotillin-2 (B) promoter deletion constructs. Flotillin promoter fragments of different length were cloned into pGL3-basic vector and transiently transfected into Hela cells. The cells were lysed 48 h post-transfection. Relative luciferase activity of the longest promoter construct was set as 1. Values are means ± standard deviation of at least 4 experiments, each measured in duplicates. *** p<0.001; **p<0.01; *p<0.05 vs. the longest construct.

## Materials and Methods

### Ethics statement

Ethical approval was not required for this study because under the German Animal Welfare Act, any procedure that involves the euthanasia of the animal and post-mortem retrieval of organs (including the uterus with the embryos) is not subject to a special ethical approval. All procedures used in this study involving embryonic fibroblast isolation have been declared to the local authorities of Regierungspräsidium Giessen (Regional Council of Giessen) (registry number V54-19c20/15cGI20/2). The mice used for the MEF isolation were treated in accordance with the recommendations of the Guide for the Care and Use of Laboratory Animals of the National Institutes of Health and of local authorities. All animals were sacrificed after proper anesthesia to ensure minimal suffering.

**Table 2:** Pairwise similarities of promoter regions (shown as % identical nucleotides).

	Flotillin-1				Flotillin-2			
	Mus Musculus	Rattus Norvegicus	Pan Troglodytes	Bos Taurus	Mus Musculus	Rattus Norvegicus	Pan Troglodytes	Bos Taurus
Homo Sapiens	51%	55%	98%	57%	58%	64%	98%	57%
Mus Musculus		83%	47%	37%		85%	55%	42%
Rattus Norvegicus			50%	39%			57%	36%
Pan Troglodytes				56%				57%

### Cell culture

HeLa cells (human cervix carcinoma, obtained through American Type Culture Collection), and primary murine embryonic fibroblasts (MEF) were cultured in Dulbecco's modified Eagle's medium (DMEM, Gibco, Invitrogen, Karlsruhe, Germany), high glucose, supplemented with 10% fetal calf serum (FCS, Gibco), 100 U/ml Penicillin and 100 µg/ml Streptomycin (Sigma-Aldrich, Taufkirchen, Germany) at 8% CO_2_ and 37°C. Cell culture medium for MEFs was additionally supplemented with 1% non-essential amino acids and 1 mM sodium pyruvate. MEFs were isolated from day E13.5 mouse embryos (strain C57BL6/J) using standard protocols (see http://www.molgen.mpg.de/~rodent/MEF_protocol.pdf).

**Table 3:** Number of putative transcription factor binding sites in flotillin promoter regions.

# of matches	Flotillin-1 promoter					Flotillin-2 promoter				
Transcription factors	Homo Sa-piens AB088101	Mus Muscu-lus NC_000083.5	Rattus Norve-gicus NC_005119.2	Pan Troglo-dytes NC_006473.2	Bos Taurus NC_007324.4	Homo Sa-piens NC_000017.10	Mus Muscu-lus NC_000077.5	Rattus Norve-gicus NC_005109.2	Pan Troglo-dytes NC_006484.2	Bos Taurus NC_007317.4
**CTCF** CTCF and Boris gene family	8	7	5	8	6	11	7	6	8	9
**DMRT** DM domain-containing transcription factors	1	2	2	1	1	1	7	4	1	1
**E2FF** E2F-myc activator/cell cycle regulators	5	7	7	5	3	5	6	4	3	4
**EGRF** EGR/nerve growth factor induced protein C & related factors	18	21	9	16	11	6	2	2	6	13
**ETSF** human and murine ETS1 factors	7	12	14	6	8	6	7	5	4	8
**GATA** GATA binding factors	1	2	2	1	1	2	3	3	2	1
**HAND** Twist subfamily of class B bHLH transcription factors	1	1	1	1	2	2	2	2	2	3
**HDBP** Huntington's disease gene regulatory region	1	1	1	1	2	2	2	1	2	3
**INSM** Insulinoma associated factors	3	4	3	3	2	1	1	1	1	2
**KLFS** Krüppel like transcription factors	13	24	15	11	12	8	2	1	8	13
**MAZF** Myc associated zinc fingers	8	12	8	8	4	2	3	3	2	8
**MOKF** Mouse Krueppel like factor	2	2	3	2	1	1	2	2	1	1
**MYBL** Cellular and viral myb-like transcriptional regulators	2	4	1	1	3	3	2	2	2	4
**PAX5** PAX-5 B-cell-specific activator protein	3	6	3	3	1	4	2	2	1	1
**PBXC** PBX1-MEIS1 complexes	2	1	3	1	3	1	1	1	1	1
**RXRF** RXR heterodimer binding sites	8	7	8	5	6	6	3	1	5	3
**SP1F** GC-Box factors SP1/GC	14	16	8	14	11	6	3	5	4	11
**STAT** Signal transducer and activator of transcription	12	5	5	10	9	6	4	5	4	5
**ZBPF** Zinc binding protein factors	16	31	14	13	23	6	6	6	6	15

Approximately 1000 bp of each genomic sequence upstream of the ATG start codon were analyzed using the “Common TF” software (Genomatix software suite). Only transcription factor binding sites present in flotillin-1 and flotillin-2 sequences of the 5 species mentioned above are included in the table.

### Plasmids and mutagenesis

Genomic fragments directly upstream of the ATG start codon of human and murine flotillin-1 and flotillin-2 promoters were amplified by PCR from genomic DNA of HeLa and MEF cells, respectively, and cloned into the pDrive cloning vector (PCR cloning kit, Qiagen, Hilden Germany). The resulting constructs served as templates for the PCR amplification of shortened promoter fragments which were subcloned into pGL3-basic (Promega, Mannheim, Germany) using KpnI and HindIII or HindIII digestions. In the case of the flotillin-1 gene, the start codon is in the second exon, while it is in the first exon of the flotillin-2 gene. For each human flotillin gene, four promoter constructs with a length of 2600, 1330, 630, and 375 bp (flotillin-1) and 2130, 1385, 930, and 560 bp (flotillin-2) were generated.

**Figure 2:**
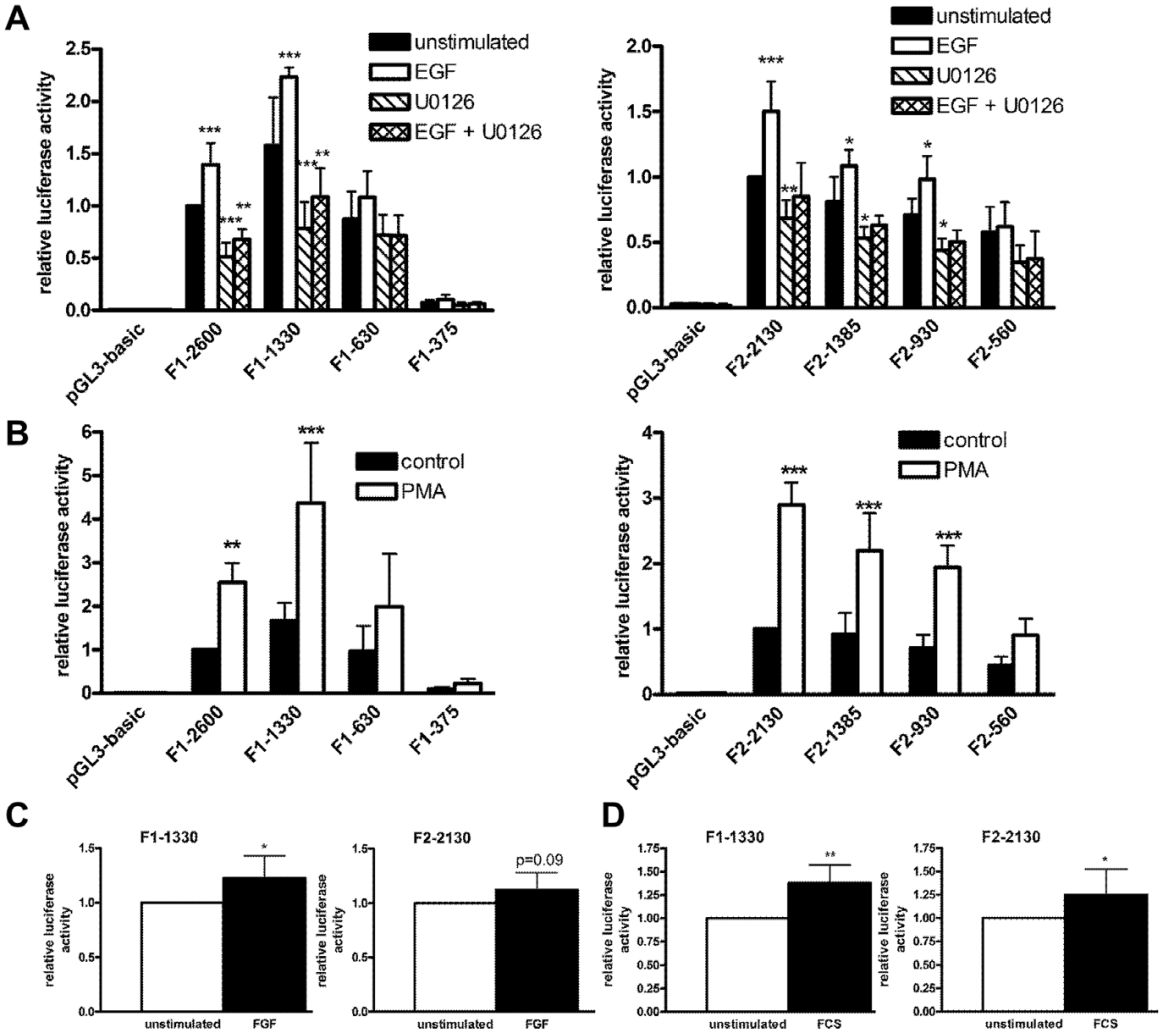
Flotillin promoter activity is induced by EGF, PMA, FGF and FCS. Flotillin promoter fragments of different length were transiently transfected into Hela cells. One day post transfection, the cells were stimulated with EGF (10 ng/ml), U0126 (10 µM) (A), PMA (10 ng/ml) (B), FGF (10 ng/ml) (C), or FCS (10%) (D) for 24 h in serum-free medium. Relative luciferase activity of the unstimulated longest promoter construct was set as 1. Values are means ± standard deviation of at least 3 experiments measured in duplicates. *** p<0.001; **p<0.01; *p<0.05 vs. unstimulated sample.

In the human genomic sequence, the flotillin-1 gene resides in the immediate vicinity of the putative gene immediate early response gene 3 (IER3). Thus, the longest flotillin-1 promoter construct F1-2660 also contains the complete sequence of IER3, while the shorter constructs either started in the 3′UTR region of IER3 (F1-1330), in the 567 bp intergenic sequence between IER3 and flotillin-1 (F1-630), or in the first exon of flotillin-1 (F1-375). The murine flotillin-1 promoter construct starts in the 3′UTR of IER3.

**Figure 3:**
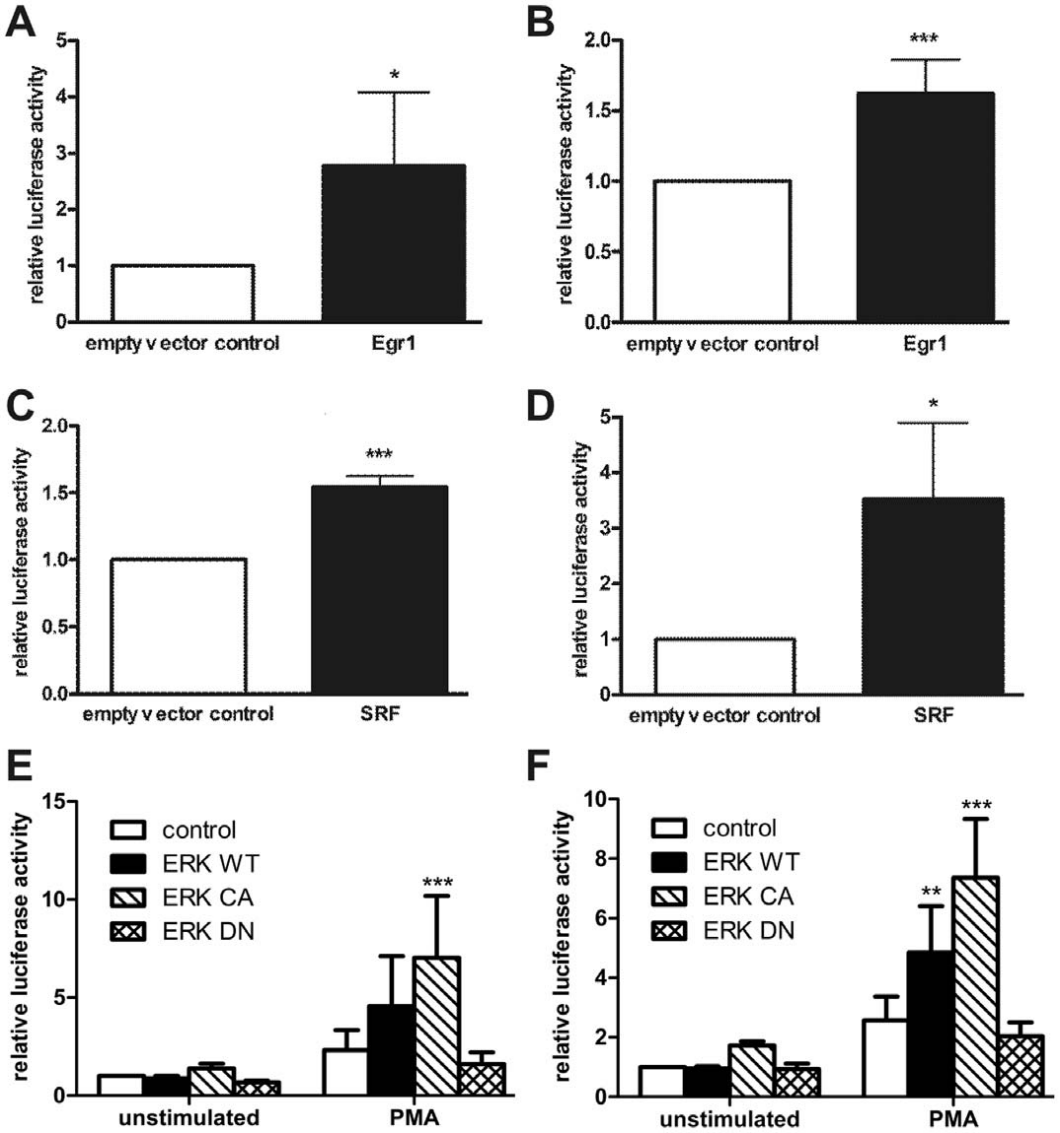
Flotillin-1 and flotillin-2 promoter activity is induced by Egr1, SRF and ERK2. Flotillin promoter constructs F1-1330 (A, C, E) or F2-2130 (B, D, F) were cotransfected into Hela cells together with expression plasmids for Egr1, SRF, empty PSV control plasmid or with the ERK2 expression constructs, and the cells were lysed 48 h post-transfection. Relative luciferase activity of the control sample was set as 1. Values are mean ± standard deviation of at least 4 experiments measured in duplicates. ***p<0.001; **p<0.01; *p<0.05 vs. control.

Similarly, the human flotillin-2 gene is flanked by a gene for the dehydrogenase/reductase family member 13 (DHRS13). The two longest flotillin-2 promoter constructs F2-2130 and F2-1385 contain parts of the coding region of DHRS13, while the two shorter constructs F2-930 and F2-560 start in the 3′UTR of DHRS13. The murine flotillin-2 promoter construct starts in the last exon of the putative DHRS13 gene.

**Figure 4:**
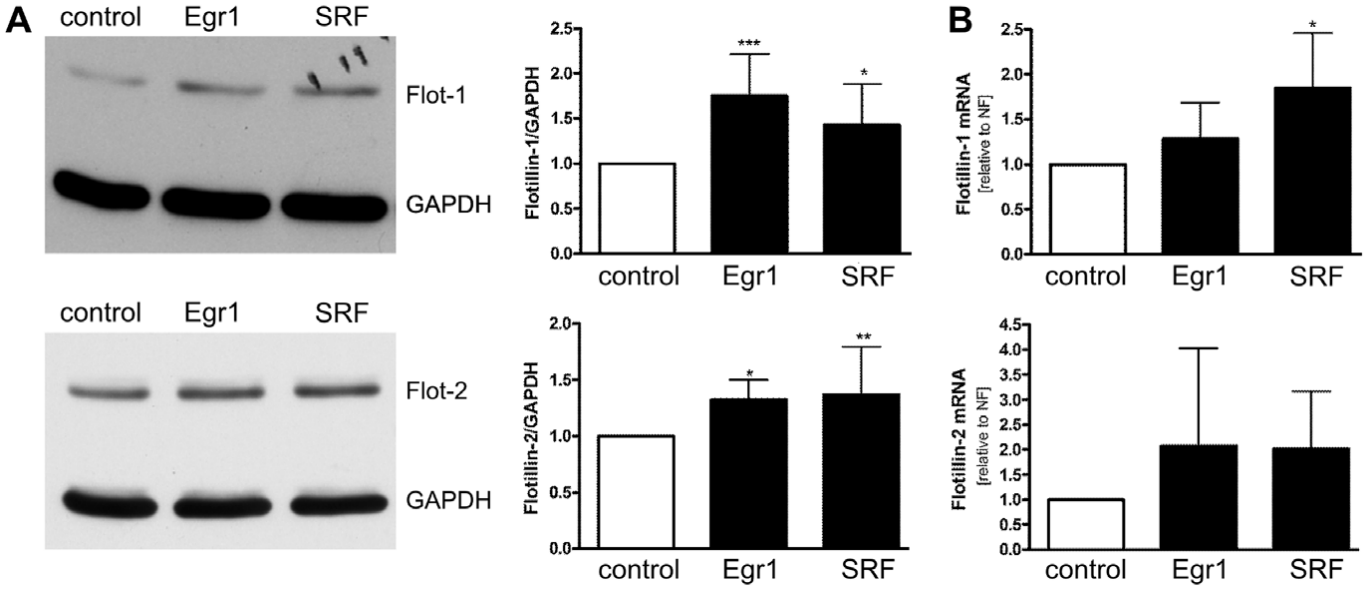
Induction of endogenous flotillin expression by Egr1 and SRF. Hela cells were transiently transfected with expression constructs for Egr1 or SRF. Empty PSV vector served as control. (A) Cell lysates were analyzed for flotillin-1 and -2 by Western blotting (left) and quantified (right). (B) RNA was isolated, transcribed into cDNA and analyzed by qPCR. Values are means ± standard deviation of 7 (Western Blot) or 4 (qPCR) experiments.*p<0.05, **, p<0.01 vs. control.

Mutagenesis of the RXR binding sites in the mouse flotillin-2 promoter were performed by means of overlap extension PCR [31] using the primers shown in Table 1. Egr1 binding sites were deleted by PCR. We generated constructs containing mutations of one of the three RXR binding sites or mutations combined with a deletion in the 3′ region that removes the Egr1 binding sites. Correctness of all constructs generated for this study was verified by sequencing.

**Figure 5:**
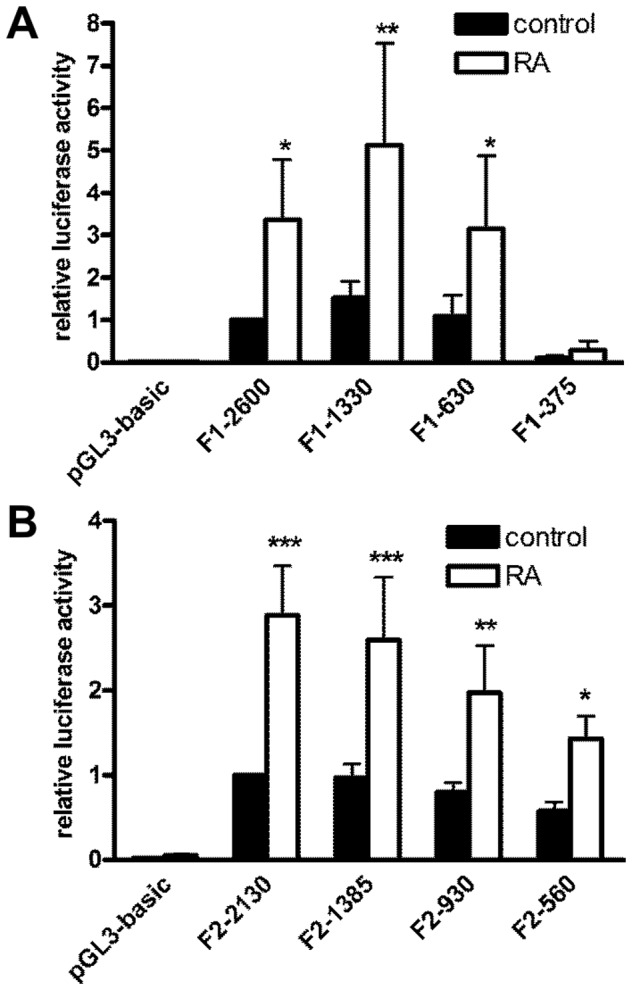
Flotillin-1 and flotillin-2 promoter activity is induced by trans-retinoic acid. Flotillin promoter fragments of different length in pGL3-basic were transiently transfected into Hela cells. One day post-transfection, the cells were stimulated with trans-RA (1 µM) for 24 h in serum-free medium. Relative luciferase activity of the unstimulated longest promoter construct was set as 1. (A) flotillin-1, (B) flotillin-2. Values are means ± standard deviation of at least 3 experiments measured in duplicates. ***p<0.001; **p<0.01; *p<0.05 vs. unstimulated sample.

Expression plasmids for RARα (#16287), RXRα (#8882), Egr1 (#11729), SRF (#11977), and PPARγ (#8895) were obtained from Addgene. (Numbers in parentheses refer to the respective plasmid numbers of Addgene). As an empty vector control, PSV-Sport (Invitrogen) was used, whereas pRL-TK (Promega, Mannheim, Germany) was utilized for the normalization of firefly luciferase activity. The expression constructs for ERK2 were obtained from N. Ahn (Univ. of Colorado, USA) and were as described [32]. All plasmids and primers are listed in Table 1.

**Figure 6:**
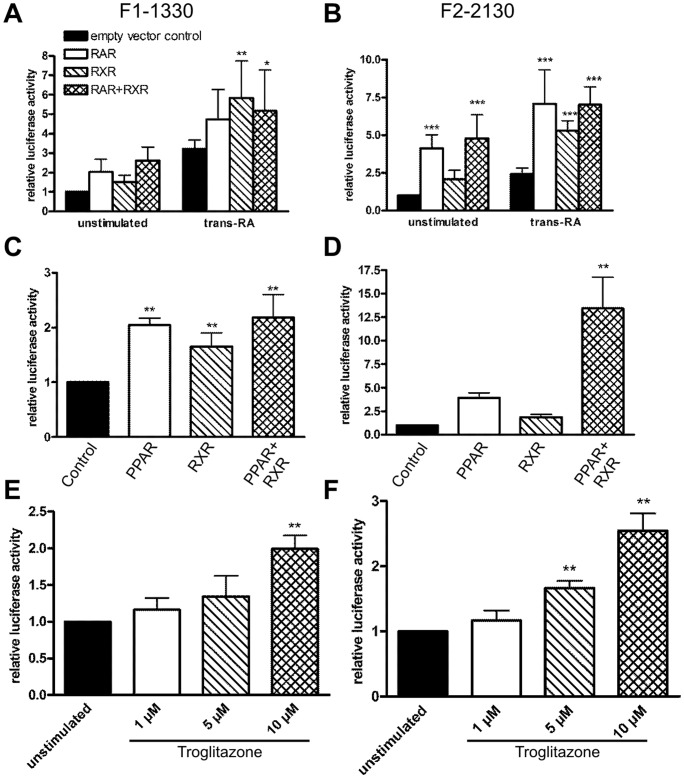
Flotillin promoter activity is induced by RXR, RAR, PPARγ, and the PPARγ activator troglitazone. Flotillin promoter constructs F1-1330 (A, C) or F2-2130 (B, D) were cotransfected into Hela cells together with expression plasmids for RAR, RXR, PPARγ or with empty PSV control plasmid. One day post-transfection, the cells were stimulated with trans-RA (1 µM) for 24 h in serum-free medium. Relative luciferase activity of the unstimulated control sample was set as 1. F1-1330 (E) and F2-2130 (F) transfected Hela cells were stimulated with troglitazone for 24 h in serum-free medium. Values are mean ± standard deviation of at least 3 experiments measured in duplicates. ***p<0.001; **p<0.01; *p<0.05 vs. respective control.

### Computer-based sequence analysis

Analysis of the flotillin-1 and flotillin-2 promoter regions of *homo sapiens, mus musculus, rattus norvegicus, pan troglodytes* and *bos taurus* for putative transcription factor binding sites common to all five species was performed using the MatInspector program. Multiple sequence alignments were done with DiAlign professional TF (Genomatix Software GmbH, Munich) [33].

**Figure 7:**
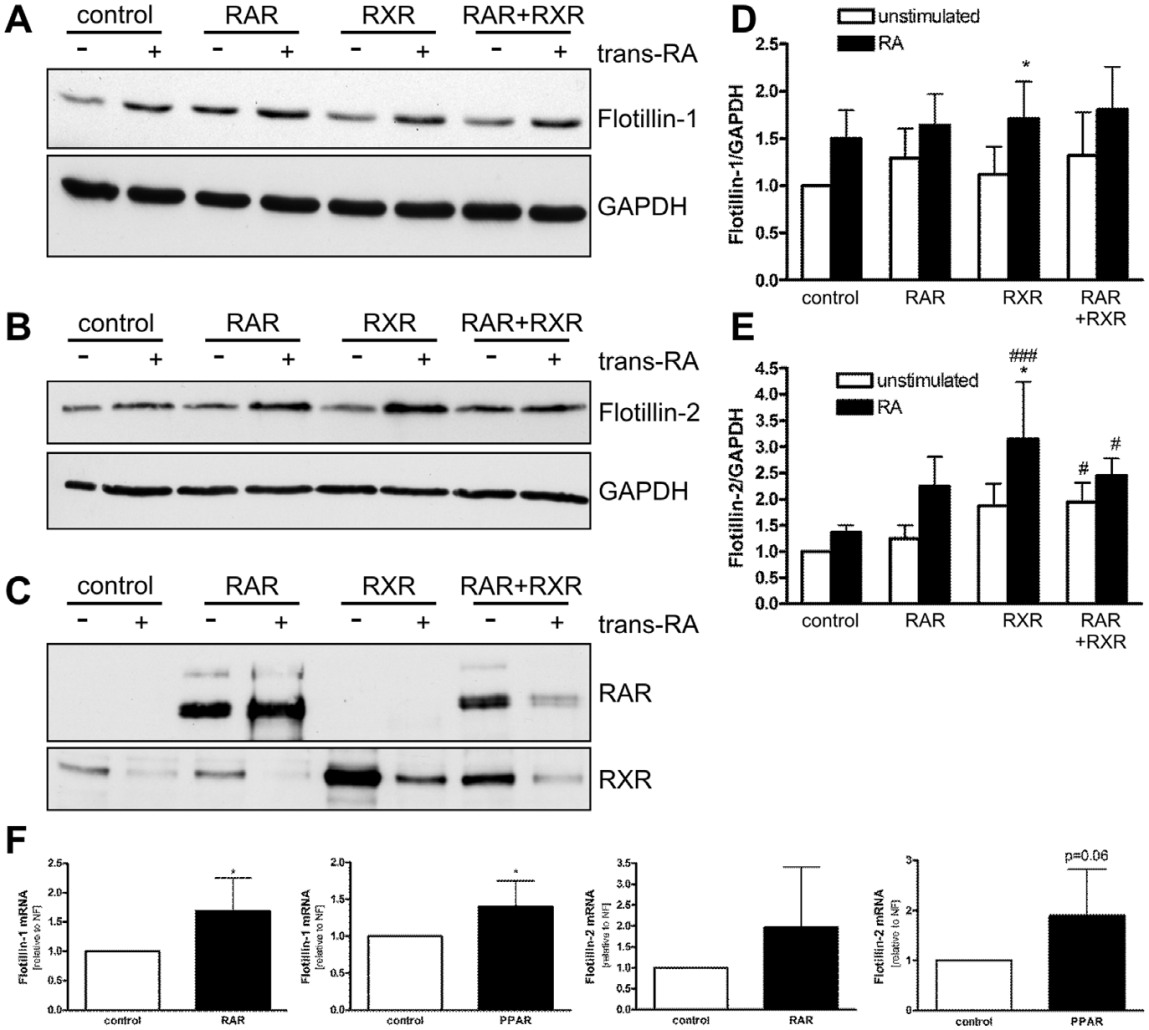
Induction of endogenous flotillin expression by RAR, RXR, retinoic acid, and PPARγ. Hela cells were transiently transfected with expression constructs for RAR, RXR, or a combination of both. Empty PSV vector served as a control. One day post-transfection, the cells were stimulated with trans-RA (1 µM) in serum-free medium for 24 h. Cell lysates were analyzed for flotillin-1 (A), flotillin-2 (B), RAR and RXR (C) by Western blotting. D and E show a densitometric quantification of flotillin expression. F: Cells were transfected with RAR or PPARγ expression construct or empty PSV. RNA was isolated, transcribed into cDNA and flotillin mRNA was measured by qPCR. Values are mean ± standard deviation of at least 3 experiments. ###, p<0.001; #, p<0.05; vs control *, p<0.05 vs. unstimulated sample.

### Transfection and reporter gene assays

24 h prior to transfection, cells were seeded to 24-well plates. For transfections, 15 ng of pRL-TK and either 150 ng promoter construct alone or 100 ng promoter construct and 100 ng expression plasmid were transfected using Lipofectamine 2000 (Invitrogen) according to the manufacturer's protocol. After 6 h, the medium was exchanged into serum-free medium. Treatment with hEGF (10 ng/ml), *trans*-retinoic acid (1 µM), phorbol myristate acetate (PMA, 10 ng/ml), MEK1/2 inhibitor U0126 (10 µM; Cell Signaling, Frankfurt, Germany), bFGF (10 ng/ml, Peprotech, Germany), FCS (10%), or troglitazone (1–10 µM, Sigma-Aldrich) in serum-free medium was started 24 h after transfection and continued for 24 h. In experiments without stimulation, cells were harvested 48 h after transfection. Cells were lysed in passive lysis buffer (Promega). Determination of firefly and renilla luciferase activity was performed with a Tecan infinite M200 plate reader, using 20 µl of lysate and 85 µl of beetle or renilla juice reagent (PJK, Kleinblittersdorf, Germany). Relative luciferase activity was calculated by dividing firefly luciferase activity by renilla luciferase activity.

**Figure 8:**
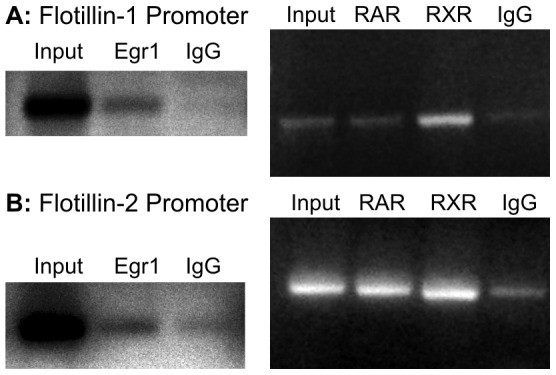
RXR, RAR, and Egr1 bind to the promoter of flotillin-1 and flotillin-2. Chromatin immunoprecipitations were carried out in Hela cells as described in Materials and Methods. DNA/protein complexes were precipitated with antibodies against RXR, RAR, Egr1 or HRS (IgG control). Coprecipitated DNA fragments were amplified with primers specific for approximately 500 bp (flotillin-1) (A) or 400 bp (flotillin-2) (B) of genomic DNA directly upstream of the ATG start codon. Results are representative of two independent experiments.

### Antibodies

Rabbit polyclonal antibodies against RARα (sc-551) and RXRα (sc-774) were purchased from Santa Cruz Biotechnology (Santa Cruz, CA, USA). Rabbit monoclonal anti-Egr1 antibody was from Cell Signaling. A mouse monoclonal antibody against GAPDH was obtained from Biozol (Eching, Germany). For detection of flotillin-1 and flotillin-2 in Western blots, monoclonal antibodies from Transduction Laboratories (Franklin Lakes, NJ, USA) were used. Secondary antibodies goat anti-mouse and goat anti-rabbit coupled to horseradish peroxidase (HRP) were obtained from Southern Biotechnologies (Birmingham, AL, USA) and Zymed (Invitrogen, Karlsruhe, Germany), respectively.

**Figure 9:**
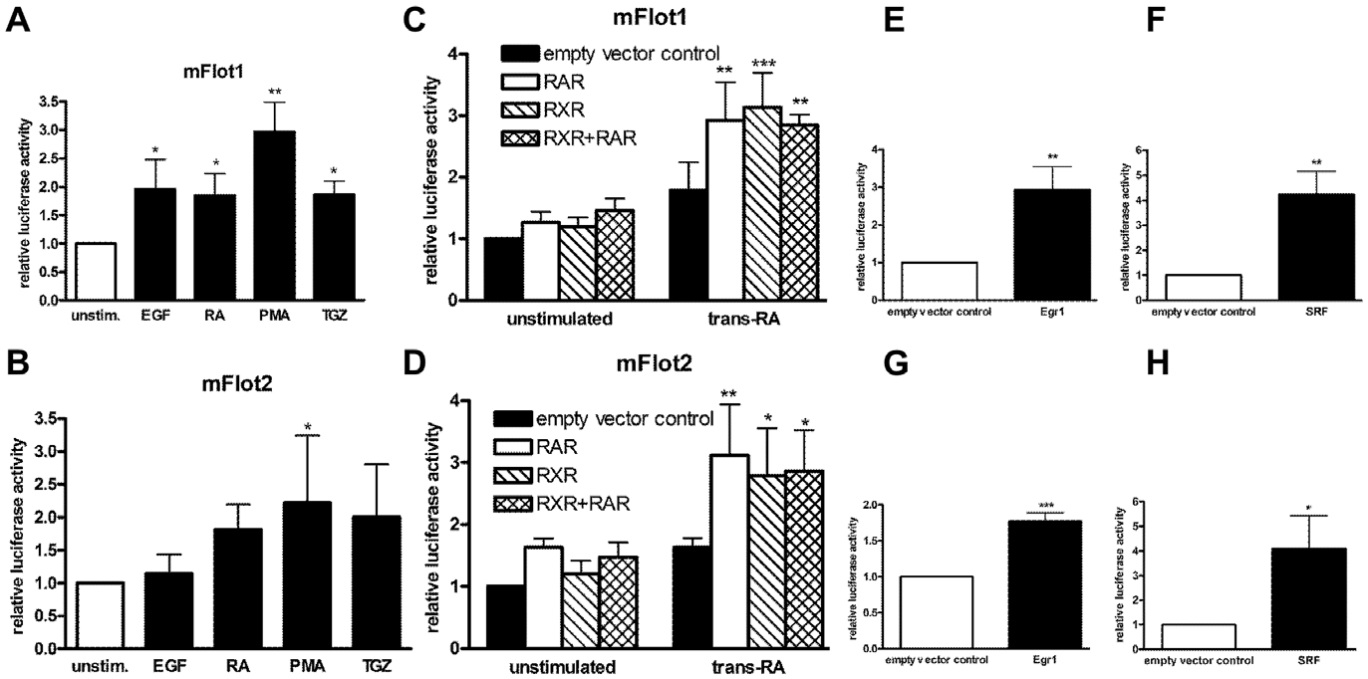
Murine flotillin promoters are activated in a similar manner as the human promoters. Murine flotillin-1 (A, C, E, G) or flotillin-2 (B, D, F, H) promoter constructs were transiently transfected into Hela cells, either alone or in combination with the expression constructs for transcription factors. One day post-transfection, the cells in A–D were stimulated with EGF (10 ng/ml), trans-RA (1 µM), PMA (10 ng/µl), or troglitazone (10 µM) in serum-free medium for 24 h. Cells in E–H were lysed 48 h post-transfection. Values are means ± standard deviation of at least 3 experiments measured in duplicates. ***, p<0.001; **, p<0.01; *, p<0.05 vs. control.

### Western blotting analysis

The same lysates that were used for determining the activity of firefly and renilla luciferases were used for Western blot analysis. Protein concentrations were determined according to Bradford using the BioRad Protein Assay reagent (BioRad, Munich, Germany). Equal protein amounts were analyzed by 10% SDS-polyacrylamide gel electrophoresis and Western blot. For antibody staining, the membranes were blocked with 5% non-fat dry milk in TBST (10 mM Tris, 150 mM NaCl, 0.05% Tween 20) and incubated overnight at 4°C with the indicated primary antibodies diluted in 2.5% non-fat dry milk in TBST. This was followed by incubation with HRP-conjugated secondary antibodies in TBST for 1 h at room temperature before development with the Amersham^TM^ ECL^TM^ Western Blotting Detection Reagents (GE Healthcare, Buckinghamshire, UK).

**Figure 10:**
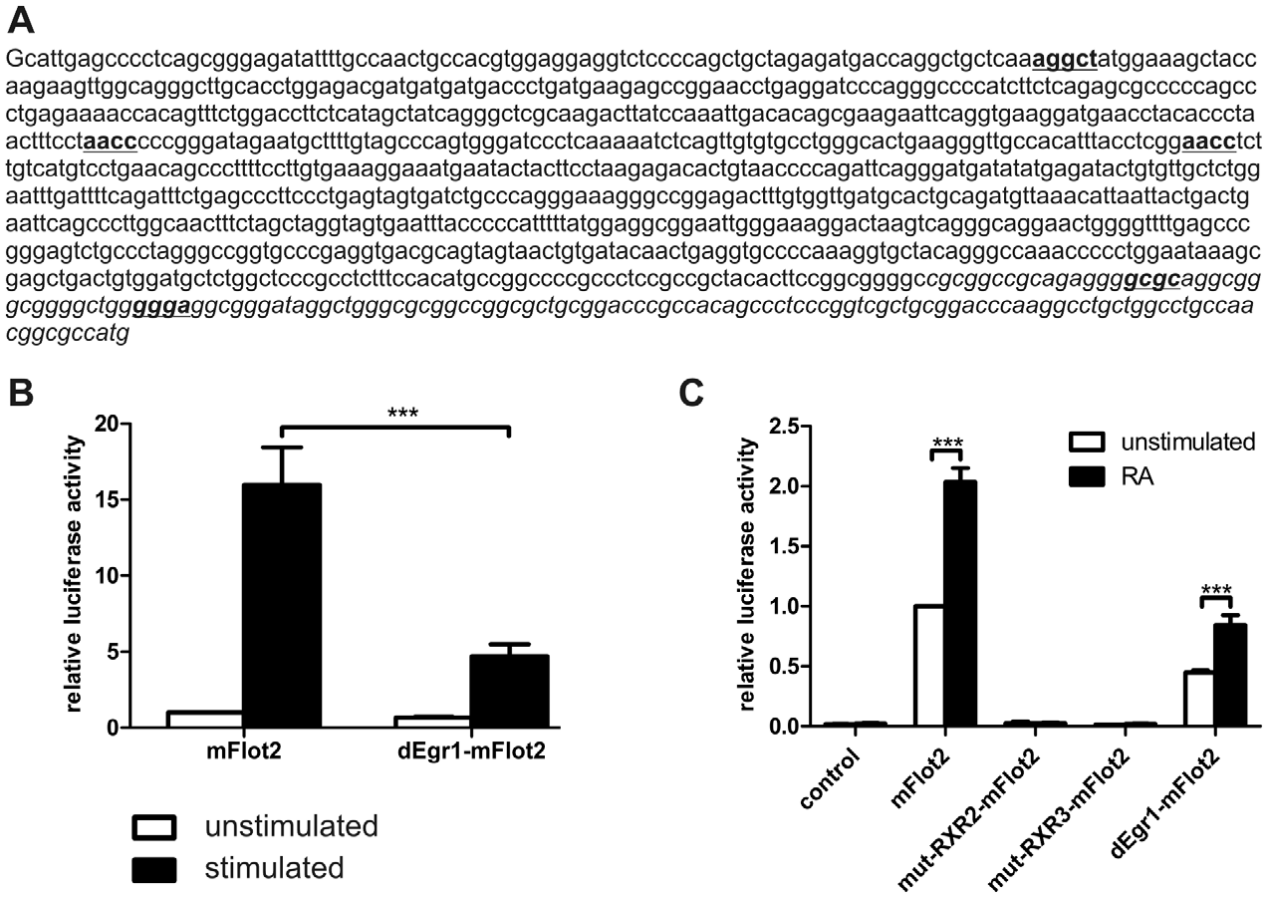
Mutagenesis of Egr1 or RXR binding sites prevents transcriptional activation of mouse flotillin-2. A: mouse flotillin-2 proximal promoter sequence. The three putative binding sites for the RXR family and the two sites for Egr1 are shown in bold and underlined. The sequence shown in italics was deleted to remove the Egr1 binding sites. Mouse flotillin-2 promoter activity after PMA stimulation (B) or retinoic acid stimulation (C). Transfection and stimulation were performed as in Figure 9.

### RNA isolation and qPCR

Total RNA was isolated using peqGold Trifast reagent (Peqlab, Erlangen, Germany). RNA (3 µg) was reverse transcribed with 150 fmol oligo(dT)15 primers and 180 U Moloney murine leukemia virus reverse transcriptase (Promega) in a total volume of 45 µl. Real time PCRs (ABI7300, Applied Biosystems, Darmstadt, Germany) were performed in triplicates with 1 µl of 5-fold diluted cDNA in 25 µl reaction mixtures using Absolute QPCR Sybr Green Mix (Thermo Fisher Scientific, Bonn, Germany). The annealing temperature was 60°C for all PCR reactions. Primers were designed to be specific for the cDNA with PerlPrimer. Primer sequences are listed in Table 1. PCR products were quantified with the ΔCt-method. The mean of the reference genes Rpl13a and GAPDH was used for normalization.

### Chromatin Immunoprecipitation (ChIP)

ChIP was performed as described [34] with slight modifications. In short, per experiment, one confluent 15 cm dish of HeLa cells was fixed in 1% formaldehyde at 37°C for 10 min. Cross-linking was stopped by addition of 125 mM glycine in PBS for 10 min at room temperature. Cells were washed with PBS and harvested by scraping. After centrifugation (5 min, 380 g, 4°C) the cell pellet was resuspended in 1,5 ml of cold lysis buffer A (10 mM HEPES, 10 mM KCl, 1.5 mM MgCl2, 0.1 mM EDTA, 0.35 M sucrose, pH 7.9) supplemented with protease inhibitor cocktail (Sigma-Aldrich) and incubated on ice for 10 min. Thereafter, the lysates were centrifugated for 15 min, 4000 g, 4°C. The obtained pellet containing the nuclei was resuspended in 1 ml cold lysis buffer B (20 mM HEPES, 420 mM NaCl, 1.5 mM MgCl_2_, 0.1 mM EDTA, 10% glycerol, pH 7.9) supplemented with protease inhibitor cocktail. The chromatin was fragmented to an average length of 200–1000 bp by sonicating 10×20 s, 25% amplitude (UW mini20, Bandelin electronic, Berlin, Germany). After centrifugation (20 min, 16000 g, 4°C), a 100 µl aliquot was removed as an input sample. The remaining chromatin solution was diluted 10-fold in ChIP-buffer (50 mM Tris, 150 mM NaCl, 0.25 mM EDTA, 1% Triton X-100, 0.1% sodium desoxycholate, pH 8.1) supplemented with 1 mM phenyl-methyl-sulfonylfluoride. The samples were precleared for 1 h at 4°C with 20 µl Pansorbin cells (Calbiochem, Darmstadt, Germany) that had been blocked with BSA (0.1%) and 0.4 mg/ml herring sperm DNA overnight and washed in dialysis buffer (50 mM Tris, 2 mM EDTA, pH 8). One third of each sample was incubated overnight with 3 µg of antibodies against RAR, RXR or with an isotype-matched immunoglobulin G (anti-HRS). For Egr1-ChIP, half of the sample was incubated with 5 µl of Egr1 antibody and the other half with 5 µl of an isotype-matched immunoglobulin G (anti-EGFR). Immunoprecipitation was performed by adding 30 µl of preblocked Pansorbin cells for 2 h at 4°C. Pansorbin beads were washed twice with ChIP-buffer and twice with PBS with centrifugation steps (2 min, 1500 g, 4°C) in between. Elution was done twice (15 min, room temperature) in 150 µl elution buffer (0.1 M NaHCO_3_, 1% SDS). Cross-linking was reversed by adding NaCl to a final concentration of 0.3 M and 20 µg of RNAse A and incubation at 65°C for 4 h. Proteinase K digestion was performed for 1 h at 55°C. Samples were purified by phenol-chloroform extraction and ethanol precipitation. PCR was performed with primers Flot1-375 fwd, Flot1-rev and Flot2-ChIP fwd and rev, respectively. PCR products were separated on 1.5% agarose gels.

### Statistical analysis

Unless otherwise stated, all experiments were performed at least three times. Data are expressed as mean ± SD. Statistical comparisons between groups were made using Student's *t*-tests, 1way or 2way ANOVA, as appropriate (GraphPad Prism 5, San Diego, CA, USA). Western blot bands were quantified by scanning densitometry using Quantity One Software (Bio-Rad) and normalized against GAPDH. Values of *p*<0.05 were considered significant (*) while values of *p*<0.01 were considered very significant (**) and *p*<0.001 extremely significant (***).

## Results

### The genomic regions of flotillin-1 and -2 upstream of the ATG start codon contain an active promoter

Four human flotillin promoter fragments with a maximum length of 2600 bp (flotillin-1) and 2130 bp (flotillin-2) were generated by PCR and cloned into the reporter gene plasmid pGL3-basic. All constructs displayed a relative luciferase activity that was significantly higher than the activity of the empty control plasmid pGL3-basic (Fig. 1). In the case of flotillin-1, the basal promoter activity was highest for the second-longest construct F1-1330 and very low for the shortest construct F1-375, indicating that the region upstream of 375 bp from the ATG start codon contains elements essential for the basal flotillin-1 promoter activity. Flotillin-2 promoter activity was found to be highest with the longest construct and was reduced to about 50% in the shortest construct reaching 560 bp upstream of ATG.

### The promoter regions of flotillin-1 and flotillin-2 are conserved among species and contain a similar set of putative transcription factor binding sites

After the identification of a functional promoter region in the human flotillin-1 and -2 genes, genomic sequences of flotillin-1 and flotillin-2 of five different species (human, mouse, rat, chimpanzee, and cow) were compared to identify conserved regions and putative transcription factor binding sites, which would imply that common transcriptional regulatory mechanisms exist. Of each genomic sequence, 1000 bp upstream of the ATG start codon were used for the computer-based analyses. The analysis was done with genomic sequences of five species to reduce the amount of false-positive matches. Sequences of mouse, rat, chimpanzee and cow were to >51% (flotillin-1) and >57% (flotillin-2) identical to the human sequence (Table 2). Analysis for putative transcription factor binding sites revealed a set of 19 transcription factor families whose binding sites are present in both flotillin-1 and flotillin-2 of all five species analyzed (Table 3). For many of them (e.g. EGRF family), the putative responsive element was found multiple times per sequence, making a functional role of these transcription factors likely. In further parts of this study, we focused on the transcription factor families EGRF, ETSF, and RXRF, the functionality of which was analyzed in more detail.

### Flotillin-1 and flotillin-2 promoters are activated by EGF in a MAPK/ERK1/2 dependent manner and by PMA, FGF and FCS

We recently identified flotillin-1 as a key regulator of EGF receptor and the downstream mitogen activated protein kinase (MAPK) ERK1/2 signaling. In this cascade, flotillin-1 forms a molecular complex with EGFR, but also with CRAF, MEK1, ERK1/2 and KSR1, thus putatively working as a novel MAPK scaffolder [13]. Because of the essential role of flotillin-1 in EGF receptor signaling, we analyzed if the expression of flotillins themselves is regulated by growth factors such as EGF. For this, the luciferase reporter assay with flotillin promoter constructs was performed in EGF stimulated cells. In the case of flotillin-1, constructs F1-2600 and F1-1330 were significantly activated by EGF as compared to the unstimulated cells, whereas the shorter ones showed no significant activation (Fig. 2A, left). Similarly, the 3 longest promoter constructs of flotillin-2 were significantly activated by EGF (Fig. 2A, right). Interestingly, the three longest flotillin-1 and all four flotillin-2 constructs displayed a basal activity even in unstimulated cells, as compared to the cells transfected with the control vector. To check if the EGF induced increase in the flotillin promoter activity was due to activation of the MAPKs ERK1/2, we used an inhibitor specific for the MAPK kinases MEK1/2, which reside immediately upstream of ERK1/2 in the pathway and are essential for ERK1/2 activation. Incubation of the cells with the MEK1/2 inhibitor U0126 not only completely abolished the stimulatory effect of EGF, but also significantly suppressed the basal promoter activity. This indicates that already the basal expression of flotillins is in part dependent on the MAPKs ERK1/2. This effect was particularly strong for flotillin-1, which itself modulates MAPK signaling. Based on the above data, flotillins thus can be added to the list of ERK1/2 regulated genes.

To check if the flotillin promoters also respond to other growth factors and stimuli, the above experiments were repeated with PMA, FCS or basic Fibroblast Growth Factor (bFGF) as stimulating agents which all result in downstream ERK1/2 activation. PMA is known to activate protein kinase C (PKC), a Ser/Thr kinase family with a role in cell growth, proliferation and tumorigenesis [35]. Activation of PKC leads to activation of cRaf and, hence, to a downstream activation of ERK1/2. Stimulation with PMA resulted in a strong activation of the three longest flotillin-1 promoter constructs and of all four flotillin-2 promoter constructs (Fig. 2B), very similarly to EGF. Since the second longest flotillin-1 and longest flotillin-2 promoter construct gave the strongest activation in the above experiments, we used these two constructs in most of the further studies. Stimulation of the cells with either bFGF which activates ERK1/2 and PI3K through the FGF receptor activation (Fig. 2C) or with FCS (Fig. 2D), which contains a mixture of growth factors, also resulted in an increased promoter activity which was more pronounced for flotillin-1 than for flotillin-2. In fact, a significant activation by FGF was only observed with flotillin-1 whereas flotillin-2 activation remained just below the significance level (p = 0.09). However, FCS-induced activation was significant for both flotillins. In summary, the promoters of both flotillins are activated by several stimuli that all derive from an upstream activation of ERK1/2.

### Flotillin-1 and flotillin-2 promoter activity and endogenous flotillin expression are increased by Egr1 and SRF

Activation of the MAPK ERK1/2 leads to downstream activation of several transcription factors that transduce the signal into the nucleus. The identification of ERK1/2 as a regulator of basal and growth factor induced flotillin promoter activity prompted us to identify the transcription factors that are responsible for the observed effects. According to the computer-based sequence analysis, candidate transcription factors include the early growth response gene Egr1 and transcription factors of the ETS family, such as Elk1. Elk1 forms heterodimers with the serum response factor SRF and binds to the serum response element in the promoter region of its target genes. Both Egr1 and SRF were able to significantly activate the promoters of flotillin-1 and flotillin-2 (Fig. 3A-D), confirming a role for these transcription factors in the transcriptional control of flotillins.

To test this further, we performed cotransfection experiments to measure the effect of ERK2 expression on flotillin promoter activation. For this, wild-type (WT), constitutively active (CA) and dominant negative (DN) mutant ERK2 expression constructs were used [32]. As shown in Fig. 3E–F, expression of WT or CA but not of DN ERK2 led to a significantly higher stimulation of flotillin transcription in PMA stimulated cells as compared to the control cells cotransfected with an empty expression vector.

To study if the increase in promoter activity is reflected by an increase in mRNA and protein content, expression of endogenous flotillins was analyzed in cells overexpressing either Egr1 or SRF, the expression of which was confirmed by Western blot in all experiments (data not shown). Although HeLa cells express fairly high amounts of flotillins already at basal conditions and their upregulation may thus be difficult to observe, overexpression of either Egr1 or SRF resulted in a significant induction of flotillin expression on protein level (Fig. 4A). RNA levels (Fig. 4B) showed the same tendency but did not reach statistical significance (except for SRF in flotillin-1 regulation) due to high variations between the experiments.

### Flotillin-1 and flotillin-2 promoter activity and endogenous flotillin expression are increased by retinoic acid, RXRα, RARα, and PPARγ

The computer-based analysis of the two flotillin promoters revealed 19 conserved putative transcription factor binding sites. Of particular interest was the presence of multiple retinoid X receptor (RXR) heterodimer binding sites in both promoters. The RXR is activated by retinoic acid, a compound that has already been described to affect the expression of flotillins [36]. Therefore, reporter gene assays with flotillin promoter constructs were performed in the presence of *trans*-retinoic acid. As shown in Fig. 5, retinoic acid is a strong activator of both flotillin promoters.

RXR is the dimerization partner of several nuclear receptors, including retinoic acid receptors (RAR) and peroxisome proliferator activated receptors (PPAR). Therefore, we next tested the effect of overexpressed RXRα, RARα, and PPARγ on the activity of flotillin promoters. Overexpression of RARα and RXRα alone or together resulted in an increased promoter activity, which was even more enhanced in the presence of retinoic acid (Fig. 6A, B). In particular, RARα exhibited a strong effect on flotillin-2 promoter activity even in unstimulated cells (Fig. 6B). Similarly, PPARγ overexpression led to an increased promoter activity of flotillin-1 (Fig. 6C). In the case of flotillin-2, PPARγ was significantly effective only in the presence of its binding partner RXR (Fig. 6D). PPARγ, similarly to RAR and RXR, is a ligand-activated transcription factor, with the antidiabetic drug troglitazone being a typical PPARγ ligand. In line with the overexpression data, stimulation with increasing amounts of troglitazone resulted in a dose-dependent activation of both flotillin promoters even in the absence of PPARγ overexpression (Fig 6E, F).

To see if the above effects on promoter activity also result in an increased expression of endogenous flotillins, flotillin protein and mRNA expression were analyzed with Western blot and qPCR in retinoic acid stimulated cells overexpressing RARα, RXRα or PPARγ. In line with the promoter data, stimulation with retinoic acid led to an increase in flotillin protein expression, especially in RXRα transfected cells (Fig. 7 A–C, densitometric quantification in 7D–E). In addition, overexpression of either RARα or PPARγ resulted in an increased flotillin expression on mRNA level (Fig. 7F). In summary, both flotillin-1 and flotillin-2 contain functional binding sites for RXR and its binding partners and, hence, can be classified as retinoic acid responsive genes.

### RXRα, RARα, and Egr1 bind to the promoter of flotillin-1 and -2

To directly show that the transcription factors RARα, RXRα and Egr1 bind to flotillin promoters, we performed chromatin IP (ChIP) assays with antibodies specific for RARα, RXRα and Egr1, and isotype-matched control antibodies. PCR primers amplifying approximately 400 bp of the genomic region of flotillin-1 and flotillin-2 upstream of the ATG start codon were used for the detection of the precipitated DNA fragment (Fig. 8). RXRα and Egr1 were found to bind to promoters of both flotillins, whereas RAR only bound to flotillin-2 promoter, consistent with the overexpression data shown in Fig. 6B.

### The transcriptional regulation of flotillins is conserved among species

All experiments shown so far were performed with the human flotillin promoter constructs. According to the computer-based sequence analysis (Table 3), all transcription factors that were identified in the present study should be able to activate the flotillin promoters of the other four species. In order to confirm this hypothesis, approximately 1000 bp of the murine flotillin-1 and -2 promoters were cloned into pGL3-basic and used for reporter gene analysis. Similar to the results obtained with the human constructs, stimulation with EGF, retinoic acid, PMA, or troglitazone resulted in an increased activity of the murine promoter constructs (Fig. 9A and B). Furthermore, both murine promoters were directly activated by the transcription factors RARα, RXRα (Fig. 9C and D), Egr1 (Fig. 9E and F) and SRF (Fig. 9G and H), confirming a species-conserved transcriptional regulation of flotillins by these transcription factors.

### Mutations of the binding sites for the RXR family or Egr1 impair flotillin-2 transcription

Our data above suggested that the RXR and EGRF transcription factor families are involved in the regulation of flotillin transcription. To show that the identified binding sites for these factors in the flotillin promoters are required for the transcriptional activation, we performed site-directed mutagenesis. Since the number of the binding sites in human flotillin promoters is too high to be feasible for these studies (see Table 3), we mutated the sites in the mouse flotillin-2 proximal promoter construct which contains 2 sites for Egr1 and 3 sites for the RXR family (Fig. 10A). The RXR binding sites were individually mutated using extension overlap PCR [31], whereas the Egr1 binding sites which are located close to each other and to the 3′ end of the promoter sequence were deleted. We also produced constructs in which individual RXR site mutations were combined with a 3′ deletion. As shown in Fig. 10B, deletion of the Egr1 binding sites severely impaired PMA-mediated transcriptional activation, although it did not completely prevent it. Single mutation of the RXR family binding sites did not abrogate the transcriptional activation of the reporter constructs upon RA stimulation (Data not shown). However, when the mutations of the RXR binding sites 2 and 3 were combined with a deletion in the 3′ region removing the Egr1 binding sites, the transcriptional activation induced by retinoic acid was completely abolished (Fig. 10C). This seems not to be due to the absence of the 3′ region as such, since the Erg1 site deletion construct exhibited a significant albeit reduced transcriptional activation and lower basal activity. Interestingly, the basal activity of the RXR site mutant constructs was also drastically reduced (Fig. 10C). In summary, our data show that RXR and Egr1 families are involved in the transcriptional regulation of flotillins across the species.

## Discussion

In general, control of transcription determines whether or not a gene is expressed and also how much of it is expressed under certain conditions. Until now, the transcriptional regulation of flotillin expression is only poorly understood, despite their important functions in various signaling pathways and their altered expression in cancer and neurodegenerative diseases. In this study, we have performed an analysis of the transcriptional regulation of human and mouse flotillins, with focus on the proximal promoter regions of the respective genes. Although we here show that these regions contain important regulatory sequences that control flotillin expression, it is possible that the genomic sequences further upstream or the intron of flotillins contain additional *cis* regulatory elements that were not identified by our study.

Here we show that flotillin-1 and flotillin-2 are direct transcriptional targets of 1) growth factor-induced MAPK-signaling, mediated by Egr1 and SRF, and 2) nuclear receptors that dimerize with RXR. Importantly, we also demonstrate that this regulation is conserved among species. In the light of our recent findings that flotillin-1 is an important regulator of MAPK signaling [13], the finding that flotillin expression is controlled by MAPKs, most likely through Egr1 and SRF activation, is especially intriguing. The MAPK pathway plays a central role in a variety of signaling networks and mediates many of the intracellular actions of e.g. growth factors. It is widely accepted that aberrant signaling can lead to malignant transformation of cells and to carcinogenesis, as has been shown e.g. for overexpressed EGFR [37]. The MAPK family members extracellularly regulated kinases ERK1 and ERK2 represent targets at which signals from different growth factor receptors converge. In quiescent cells, ERK1/2 are mainly located in the cytoplasm where they are bound to MEK1/2. The nuclear export signal of MEK1/2 prevents the nuclear translocation of ERK1/2 [38]. Phosphorylation of ERK1/2 by MEK1/2 is necessary for the dissociation of the MEK/ERK complex. Since our recent data show that flotillin-1 is a novel MAPK scaffolder that is essential for full ERK activation and directly interacts with both MEK1 and ERK2 [13], it may also regulate the localization of the MEK/ERK complex. Thus, transcriptional regulation of flotillins by MAPK pathway might provide a feedback loop that affects the activation status and duration of MAPK signaling.

Activated ERK1/2 dimerize and translocate to the nucleus where they bind to and phosphorylate transcription factors such as Elk-1 [39]–[41], c-Myc [42], and c-Fos [43], which then induce the transcription of several target genes. Elk-1 is one of the best-studied ERK1/2-activated transcription factors and belongs to the group of Ets ternary complex factors (overview in [44]). Direct phosphorylation of Elk-1 at the C-terminus by ERK1/2 occurs rapidly at six sites [45]. In addition, a MAPK-independent phosphorylation of Elk-1 in response to FGF stimulation or Raf activation has been described [46]. Elk-1 forms a complex with serum response factor (SRF) and binds to the serum response element (SRE) in promoters of several immediate early genes such as c-Fos [47] or Egr1 [48]. SRF is essential for the activation through the SRE, since mutations that abolish the SRF-binding inhibit the activation of gene expression [49]. The SRF gene itself is induced in response to serum stimulation [50].

ERK1/2 are important contributors to cell proliferation, since common downstream targets of the three ERK1/2 substrates c-Fos, Elk-1, and c-Myc are genes that regulate cell cycle progression, such as cyclin D1 (summarized in [51]). In line with this, we recently found that flotillins are needed for cyclin D1 expression, since a knockdown of flotillin-1 in HeLa cells resulted in a reduced basal as well as EGF-induced cyclin D1 expression [13] and also in an arrest of the cell cycle in G1 (our unpublished observations), indicating that flotillins are needed for a sustained signaling downstream of ERK1/2. In general, activation of the Raf/MEK/ERK cascade is associated with increased proliferation, and a dysregulation thereof is a hallmark of many tumors. In fact, activating mutations of upstream components of ERK1/2 are responsible for many human cancers, as summarized in [52].

Other typical ERK1/2 downstream target genes are “early response genes” such as the transcription factor c-Fos and the early growth response gene Egr1. The promoter region of these genes contains at least one serum response element SRE (see above). Egr1, originally described as NGFI-A [53], is a zinc-finger transcription factor whose expression is low in resting cells, but is rapidly induced upon EGFR activation [54] in an ERK1/2-dependent manner [55] and after treatment with PMA, EGF or serum [56]. In this study, we show that all these stimuli also result in flotillin promoter activation, further pointing to ERK1/2 and Egr1 as important regulators of flotillin expression.

The Egr1 promoter contains five SREs [48] that are bound by Elk-1/SRF dimers. Accordingly, overexpression of SRF in HeLa cells results in an increased expression of Egr1 (our unpublished data). Egr1 controls the expression of a large number of target genes, most of which are related to cell growth and apoptosis, such as p53, TGFß1 and PTEN [57]–[59]. In addition, Egr1 downregulates its own expression [60], resulting in termination of Egr1-regulated gene expression, thus making sure that the signaling is only transient. The potential role of Egr1 in cancer is not fully understood, since Egr1 has been shown to either promote [61] or suppress cell proliferation and/or cancer progression in a cell and tissue specific manner (overview in [62]). Here we show that Egr1 overexpression results in transcriptional activation of flotillins and increase in the cellular flotillin protein amount, suggesting that flotillins are Egr1-regulated genes. Furthermore, removal of the Egr1 binding sites severely impairs the PMA induced transcriptional activation of the mouse flotillin-2 gene. Further evidence for this comes from microarray analysis of Egr1 knockout MEFs in which the expression of flotillin-1 mRNA was found to be decreased [59]. This finding was not studied further by the authors, but it is well supported by our data presented here.

The regulation of flotillin expression by Egr1 and SRF, both of which have multiple binding sites within the human flotillin promoters and are activated or induced upon ERK1/2 activation, may seem at the first glance somewhat redundant, with Egr1 being under transcriptional control of SRF. However, flotillins are not the only genes under direct control of both SRF and Egr1. Transcription of the ubiquitin carrier protein (UCP) was also shown to be directly controlled by SRF and Egr1 and to be upregulated upon treatment of HeLa cells with EGF, HGF, PMA, or serum [56], similarly to flotillins. Therefore, Egr1 and SRF can induce gene expression independently of each other as well as in collaboration.

The *in-silico* analysis of the mammalian genomic flotillin-1 and -2 sequences revealed 19 different, species-conserved putative transcription factor families, whose complete functional analysis is beyond the scope of this publication. To confirm the reliability of our computer-based analysis, another transcription factor family whose influence on the regulation of flotillin expression appeared interesting was analyzed. Independent from the regulation of flotillins by Egr1 and SRF, flotillin promoter activity and endogenous flotillin expression were increased by the vitamin A derivative all-*trans* retinoic acid, which regulates gene expression by acting as a ligand for the nuclear receptors RAR (all-*trans* retinoic acid) and RXR (the all-*trans* retinoic acid metabolite 9-*cis* retinoic acid). Mutations of the the individual binding sites for the RXR family factors in the mouse flotillin-2 promoter did not result in reduced activation, whereas a combination with a 3′ deletion removing the Egr1 binding sites completely abrogated the RA induced transcriptional response. However, since the removal of the 3′ region containing the Erg1 binding sites alone did not cause an impairment of RA induced activation, the identified RXR binding sites appear to be important for the transcriptional regulation, together with the 3′ region.

Retinoic acid exerts versatile cellular effects and regulates cell differentiation and cell growth and acts as a morphogen during embryogenesis (for review, see [63]). Furthermore, retinoic acid causes a MEK1/2 dependent phosphorylation of ERK2 and activation of Elk-1 in HL-60 myeloblastic leukemia cells [64], linking retinoid metabolism to MAPK signaling. Another link is provided by the fact that EGF stimulates the expression of retinoic acid receptor RARα [65].

The retinoid X receptor RXR serves as an obligatory binding partner for several members of the nuclear receptor superfamily including LXR, PPAR, and RAR (reviewed in [66], [67]). Due to its role as a heterodimerization partner, RXR is able to control the function of many nuclear receptors. The peroxisome proliferator activated receptors, PPARs, are activated by various lipids, and long-chain fatty acids most likely are their natural ligands [68]. PPARγ plays a critical role in adipogenesis and fat storage [69] but also in glucose metabolism and thus in energy regulation. Nuclear receptors are of great interest for drug development and some such as PPARγ are already successfully targeted by antidiabetic drugs [70]. The upregulation of flotillin expression upon retinoic acid treatment or forced expression of RAR, RXR or PPAR suggests a role for flotillins in embryogenesis, cell differentiation as well as lipid and glucose metabolism. Experimental proof for the function of flotillins in all these processes already exists. The expression of flotillins depends on the differentiation status of a cell and increases during the differentiation process of osteoclasts [71], skeletal myoblasts [72], and 3T3 fibroblasts [1]. In drosophila, flotillins are most abundantly expressed in the embryonic nervous system [73], suggesting a morphogenic function. The drosophila flotillin-2 gene appears to be under the control of the transcription factors of Grh, AP-1 and ETS families [30]. Overexpression of flotillin-2 in drosophila leads to abnormal morphogen distribution, impaired wing development, mislocalization of adhesion molecules, as well as to abnormalities in eyes, ocelli and bristles [74], [75]. However, flotillin-2 overexpressing flies are viable and fertile, but show a defect in the control of transcriptional response after embryonic wounding, implicating that flotillin-2 is important for the restriction of the transcriptional response in the vicinity of the wound [30]. In goldfish [2] and zebrafish [76], [77], flotillins are involved in axonal regeneration after injury of the optic nerve and were originally discovered due to upregulation of their expression after a lesion of the goldfish optic nerve [2]. In mammals, they contribute to neurite branching and synapse formation in cultured cells [78], [79]. However, flotillin-1 [80] and flotillin-2 (our unpublished results) knockout mice are viable, fertile, and morphologically normal. Thus, some of the phenotypes observed after flotillin depletion or overexpression in cultured cells may not directly reflect the essential nature of flotillins in multicellular organisms. On the other hand, flotillins are extremely well conserved between species that are not closely related, suggesting that they are in some subtle manner important for these organisms. In our flotillin-2 knockout mice, we have observed changes in the transcription and expression of many proteins that regulate cell proliferation, adhesion and signaling (AB and RT, unpublished data). Thus, it is also possible that the loss of function of flotillins is compensated by an increased expression of e.g. signaling proteins to overcome the defects, and no obvious phenotype is observed, different from cell culture systems. Therefore, is it is likely that the true molecular functions of flotillins still remain to be discovered, and these findings stress the importance of animal models for the verification of the functional studies in cultured cells.

In summary, we have here characterized the expression of flotillins at the level of transcription and were able to identify flotillins as transcriptional targets of MAPK signaling, mediated by the transcription factors Egr1 and SRF. Furthermore, retinoic acid, RXRα, RARα and PPARγ exert positive effects on the expression of flotillins. These finding are of major importance for understanding the function of flotillins in many vital processes of the cells, such as differentiation, hormone and growth factor signaling, regulation of cell proliferation and carcinogenesis. Our results here thus shed important light on the characterization of flotillin function not only at protein but also at the transcriptional level.
